# Pharmacokinetics, safety, and tolerability of olaparib and temozolomide for recurrent glioblastoma: results of the phase I OPARATIC trial

**DOI:** 10.1093/neuonc/noaa104

**Published:** 2020-04-29

**Authors:** Catherine Hanna, Kathreena M Kurian, Karin Williams, Colin Watts, Alan Jackson, Ross Carruthers, Karen Strathdee, Garth Cruickshank, Laurence Dunn, Sara Erridge, Lisa Godfrey, Sarah Jefferies, Catherine McBain, Rebecca Sleigh, Alex McCormick, Marc Pittman, Sarah Halford, Anthony J Chalmers

**Affiliations:** 1 Institute of Cancer Sciences, University of Glasgow, Glasgow, UK; 2 Brain Tumour Research Centre, University of Bristol, Bristol, UK; 3 Institute of Cancer and Genomic Sciences, University of Birmingham, Birmingham, UK; 4 Division of Informatics, Imaging and Data Sciences, University of Manchester, Manchester, UK; 5 Institute of Neuroscience and Psychology, University of Glasgow, Glasgow, UK; 6 Edinburgh Centre for Neuro-Oncology, NHS Lothian, Edinburgh, UK; 7 Cancer Research UK Centre for Drug Development, London, UK; 8 Cambridge University Hospitals NHS Foundation Trust, Cambridge, UK; 9 The Christie NHS Foundation Trust, Manchester, UK; 10 LGC Group, Cambridgeshire, UK; 11 AstraZeneca, Macclesfield, UK

**Keywords:** glioblastoma, olaparib, temozolomide, pharmacokinetics, blood-brain barrier, poly(ADP-ribose) polymerase

## Abstract

**Background:**

The poly(ADP-ribose) polymerase (PARP) inhibitor olaparib potentiated radiation and temozolomide (TMZ) chemotherapy in preclinical glioblastoma models but brain penetration was poor. Clinically, PARP inhibitors exacerbate the hematological side effects of TMZ. The OPARATIC trial was conducted to measure penetration of recurrent glioblastoma by olaparib and assess the safety and tolerability of its combination with TMZ.

**Methods:**

Preclinical pharmacokinetic studies evaluated olaparib tissue distribution in rats and tumor-bearing mice. Adult patients with recurrent glioblastoma received various doses and schedules of olaparib and low-dose TMZ in a 3 + 3 design. Suitable patients received olaparib prior to neurosurgical resection; olaparib concentrations in plasma, tumor core and tumor margin specimens were measured by mass spectrometry. A dose expansion cohort tested tolerability and efficacy of the recommended phase II dose (RP2D). Radiosensitizing effects of olaparib were measured by clonogenic survival in glioblastoma cell lines.

**Results:**

Olaparib was a substrate for multidrug resistance protein 1 and showed no brain penetration in rats but was detected in orthotopic glioblastoma xenografts. Clinically, olaparib was detected in 71/71 tumor core specimens (27 patients; median, 496 nM) and 21/21 tumor margin specimens (9 patients; median, 512.3 nM). Olaparib exacerbated TMZ-related hematological toxicity, necessitating intermittent dosing. RP2D was olaparib 150 mg (3 days/week) with TMZ 75 mg/m^2^ daily for 42 days. Fourteen (36%) of 39 evaluable patients were progression free at 6 months. Olaparib radiosensitized 6 glioblastoma cell lines at clinically relevant concentrations of 100 and 500 nM.

**Conclusion:**

Olaparib reliably penetrates recurrent glioblastoma at radiosensitizing concentrations, supporting further clinical development and highlighting the need for better preclinical models.

**Key Points**

1. Olaparib penetrates core and margin regions of GBM despite not crossing the intact BBB.
2. Tumor olaparib concentrations range from 100-1000 nM, which achieve radiosensitization in vitro.
3. Olaparib given three times per week can be safely combined with continuous low-dose TMZ.

**Importance of the Study**

Despite failing to cross the intact blood–brain barrier (BBB) in preclinical models, olaparib penetrated both tumor core and tumor margin regions of recurrent glioblastoma at concentrations similar to those observed in breast cancer patients. The novel observation that tumor margin concentrations of olaparib were similar to those in the tumor core indicates clinically significant enhancement of BBB permeability even in 5-aminolevulinic acid nonfluorescing regions of glioblastoma. Olaparib effectively radiosensitized 6 glioblastoma cell lines in vitro at the median tumor concentration (500 nM) and the lower end of the tumor concentration range (100 nM). While olaparib exacerbated the hematological toxicity of TMZ, as expected, intermittent dosing was tolerated. These observations support further clinical development of olaparib in glioblastoma in combination with radiation therapy and indicate that conventional preclinical models of the BBB do not predict clinical pharmacokinetics.

Improvements in outcomes for brain tumor patients have failed to match those for many extracranial cancers. Glioblastoma (GBM) is the most common malignant primary brain tumor and confers a poor prognosis, with average life expectancy being 12–18 months for patients undergoing neurosurgical resection followed by radiotherapy with concomitant and adjuvant temozolomide (TMZ) chemotherapy.^1^ Treatment options for recurrent GBM are particularly limited; recent randomized studies have yielded 6-month progression-free survival (PFS) rates of 18–35% and median overall survival durations of 9–10 months.^2,3^

Poor treatment outcomes have been attributed in part to the failure of systemic agents to penetrate brain tumors at therapeutic levels, for which the blood–brain barrier (BBB) has largely been held responsible.^4^ The BBB protects the central nervous system from toxic molecules in the blood through ultrastructural features that restrict paracellular diffusion of polar solutes, and efflux mechanisms that export substrate molecules. The human BBB expresses high levels of ATP-binding cassette transporter proteins, notably multidrug resistance protein 1 (MDR1) (also known as P-glycoprotein).^5^

Blood vessels in GBM are grossly abnormal, however, exhibiting microvascular proliferation and increased permeability,^6,7^ which cause florid contrast enhancement on magnetic resonance imaging (MRI). While BBB integrity is grossly disrupted in the contrast-enhancing “tumor core” regions of GBM, the extent to which the BBB is compromised in marginal regions, which do not display contrast enhancement, is poorly understood. Whereas most conventional cytotoxic drugs are large, polar molecules that do not penetrate GBM,^8^ the pharmacokinetic (PK) properties of “small molecule” agents are likely to be more conducive to penetration of the dysfunctional BBB. At the moment, however, there is little clinical PK data with which to substantiate this theory. Small molecule treatments for GBM have been ineffective to date,^9^ but this may reflect lack of biological efficacy as much as failure of drug penetration.^10–12^ Hence there is a need for detailed PK studies of candidate small molecule therapies in glioblastoma patients, which should characterize drug delivery to the tumor margins as well as the tumor core.

Olaparib is an orally bioavailable small molecule inhibitor of poly(ADP-ribose) polymerase (PARP), which contributes to repair of DNA damage induced by ionizing radiation (IR) and alkylating drugs, including TMZ.^13^ While single agent activity of PARP inhibitors is restricted to tumors with defects in homologous recombination DNA repair,^14,15^ olaparib and other PARP inhibitors sensitize a broad spectrum of cancer cells to both IR and TMZ and potentiate these agents in preclinical glioma models.^16^ PARP inhibitors also have vasodilatory effects that have been associated with increased drug delivery to tumors in preclinical models.^17^ Despite these promising preclinical data, clinical development of olaparib for GBM has been hampered by lack of PK information, the absence of a reliable pharmacodynamic (PD) biomarker of PARP inhibition, and the propensity of PARP inhibitors to exacerbate hematological side effects of TMZ.^18^

The aims of this study were fourfold: preclinically, to measure penetration of the normal brain and orthotopic GBM xenografts by olaparib; clinically, to characterize olaparib PK in core (5-aminolevulinic acid [5-ALA] fluorescing) and margin (5-ALA nonfluorescing) regions of recurrent GBM; to ascertain whether olaparib affects tumor perfusion and vascular permeability; and to assess the safety and tolerability of combining olaparib with a 42-day, daily low-dose TMZ regimen. This TMZ schedule was selected because it is given concomitantly with IR to patients with newly diagnosed GBM and represents the clinical “line of sight” for the combination with olaparib. Having established the range of olaparib PK in patient specimens, we evaluated the radiosensitizing effects of these clinically relevant concentrations in established and patient-derived GBM cell lines.

## Methods

### Preclinical Methods

Canine kidney epithelial cells, stably expressing human MDR1 cDNA (Madin-Darby canine kidney type II [MDCKII]–MDR1 cells) or empty vector (MDCKII control), were used to model the intact BBB and determine permeability coefficients of (^14^C)-olaparib in the presence of ketoconazole (25 μM). Whole body autoradiography was performed on male pigmented rats after single oral administration of [^14^C]-olaparib (15 mg/kg; 5 MBq/kg). For autoradiography studies, animal care and experiments were carried out in accordance with AstraZeneca guidelines, which comply with UK standards. Olaparib PK was assessed in CD1 nude mice bearing intracranial G7 GBM xenografts from which tumor, blood, and contralateral brain specimens were taken and snap frozen 2, 5, and 24 hours after dosing (50 mg/kg). Olaparib concentrations were measured by mass spectrometry. Xenograft experiments were performed under the relevant UK Home Office Project Licence and carried out with ethical approval from the University of Glasgow under the Animal (Scientific Procedures) Act 1986 and the EU directive 2010. Mice were maintained in individually ventilated cages with environmental enrichment and following the guidelines of ARRIVE (Animal Research: Reporting of In Vivo Experiments). Radiosensitizing effects of olaparib (100 and 500 nM) were measured by clonogenic survival assay in established (T98G, UVW) and patient-derived cell lines (G7, E2, G1, R10). The linear quadratic model was fitted and integrated to determine mean inactivation dose (MID) for each condition and sensitizer enhancement ratios (SERs) calculated as MID ratios between control and olaparib treatments. Ratiometric *t*-test analysis determined SER confidence intervals and *P*-values. Further details are available in the Supplementary Material.

### Clinical Study Population

OPARATIC (NCT01390571) was a phase I study investigating the pharmacokinetics, safety, and toxicity of olaparib in combination with TMZ in adult patients with recurrent GBM. Stage 1 was a pilot study to confirm tumor penetration; stage 2 was divided into dose escalation and dose expansion cohorts.

Eligible patients were aged ≥18 years with World Health Organization performance status 0–2 and had radiological evidence of recurrent GBM (Response Assessment in Neuro-Oncology [RANO] criteria) after primary treatment with chemoradiotherapy and adjuvant chemotherapy. Exclusion criteria included previous chemotherapy for recurrent GBM; radiotherapy, endocrine therapy, or immunotherapy within 12 weeks, or chemotherapy or biological therapy within 4 weeks. Study investigations and treatments were approved by an NHS Research Ethics Committee in accordance with assurances approved by the UK Medicines and Health Regulatory Authority. Informed consent was obtained from each participant and data were anonymized. Isocitrate dehydrogenase (IDH) mutational status was not available for several of the patients (recruitment commenced in 2012, before IDH testing was routinely conducted) and IDH status was not an eligibility criterion.

### Study Design and Treatments

Stage 1 patients received olaparib tablets 200 mg twice daily for 7 doses prior to resection of recurrent tumor. Stage 2 patients (dose escalation phase) received different doses and schedules of olaparib and TMZ for 42 days of each 56-day cycle using a 3 + 3 dose escalation design (Fig. 2). Up to 3 cycles were delivered in the absence of disease progression or unacceptable toxicity. Patients progression free after 3 cycles were eligible for further cycles if approved by sponsor. Neurosurgery was optional; patients undergoing resection received olaparib at the designated cohort dose for at least 3 days prior to surgery (“cycle 0”). After postoperative recovery, patients received olaparib/TMZ according to cohort. Patients not undergoing resection received cycle 0 then immediately started cycle 1 of olaparib/TMZ. Neurosurgical resection was mandatory in the expansion cohort.

### Study Assessments and Trial Endpoints

The primary endpoint of stage 1 required olaparib to be detectable above the lower limit of quantification in at least one tumor specimen from up to 6 patients. At neurosurgery, contrast-enhancing tumor regions identified on preoperative MRI scans were targeted for resection. Specimens were assessed by intraoperative cytology; 3 regions of viable, solid tumor from each patient were snap frozen for PK analysis by liquid chromatography mass spectrometry (LC-MS). In the expansion cohort, 5-ALA guided neurosurgery enabled discrimination of fluorescent “core” tissue from nonfluorescent “margin” tissue. Up to 3 tumor margin biopsies were taken from each patient, from each of which half was frozen for PK analysis and half fixed for histology. Blood and tumor specimens for PK analysis were taken 3–5 hours after the final preoperative olaparib dose.

The stage 2 dose escalation primary endpoint was maximum tolerated dose (MTD) and schedule of olaparib/TMZ. Toxicity was defined using the National Cancer Institute Common Terminology Criteria for Adverse Events version 4.02. If 2 or more of 6 patients at one dose level experienced a dose limiting toxicity (DLT), this was considered “not tolerated” and designated the maximum administered dose (MAD). Dose escalation and expansion decisions were influenced by the need for dose reductions of olaparib and TMZ as well as DLTs as defined by the protocol. MTD was defined as either reduced dose or less frequent administration of olaparib than MAD and was further evaluated in the dose expansion cohort to generate the recommended phase II dose (RP2D).

Secondary endpoints included PFS at 6 months (PFS-6, assessed radiologically using the RANO criteria) and radiological measurement of BBB permeability and perfusion by diffusion weighted and dynamic contrast-enhanced (DCE) MRI. Patients underwent 2 baseline MRI scans 24 hours apart before commencing olaparib and a third scan after at least 3 days of olaparib, prior to surgery. Further details of the imaging protocol are provided in the Supplementary Material. Confidence intervals for PFS data were calculated using the exact binomial distribution.

Exploratory endpoints included correlation of tumor core and margin olaparib concentrations with plasma levels and histological and immunohistochemical (IHC) features including hematoxylin and eosin (H&E), Ki67, PARP-1, and blood vessel density. Histopathological methods are provided in the Supplementary Material. Additional GBM specimens for pilot poly(ADP-ribose) IHC studies were obtained from Brain Tumour Bank South West (Bristol, UK) with appropriate ethical approvals.

## Results

### Preclinical Pharmacokinetic Assessment of Olaparib

**Fig. 1:**
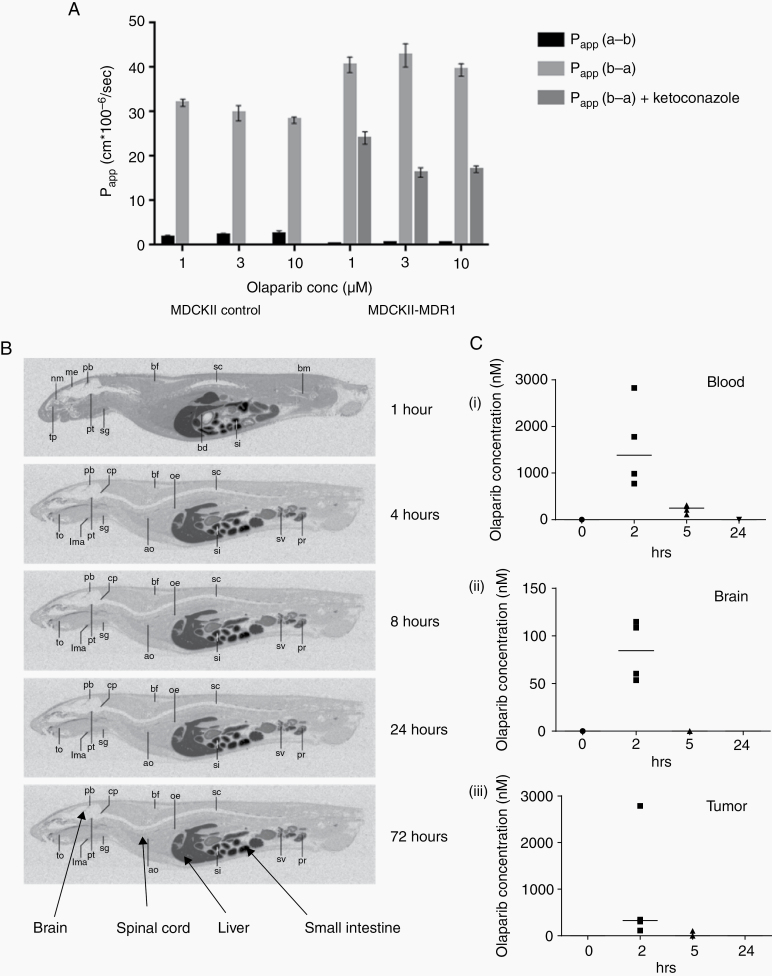
Preclinical pharmacokinetic assessment of olaparib. (A) Directional transport of olaparib across MDCKII cells stably expressing human MDR1 cDNA (MDCKII-MDR1) or empty vector (MDCKII control) was measured after 120 minutes incubation with 1, 3, and 10 µM [^14^C]-olaparib alone or with 25 µM ketoconazole. Apparent permeability coefficients were calculated for apical to basolateral (P_app_ a‒b) and basolateral to apical transport (P_app_ b‒a) as described in Methods. Olaparib efflux was shown to require expression of MDR1 and to be reduced by the MDR1 inhibitor ketoconazole. (B) Single oral doses of 15 mg/kg [14C]-olaparib were administered to male pigmented rats that were subsequently culled, sectioned, and subjected to whole body autoradiography at the timepoints shown. Radioactivity was excluded from the central nervous system in all animals at all timepoints. (C) Twelve weeks after intracranial implantation of G7 glioblastoma xenografts, CD1 nude mice were dosed with olaparib (50 mg/kg) by oral gavage. Blood, tumor, and contralateral (non tumor bearing) brain were harvested and snap frozen at the time points shown. Olaparib levels were measured by mass spectrometry (Pharmidex) and are shown as dotplots with horizontal bars representing median values (*n* ≥ 3 for each timepoint). Note different *y*-axis scales.

Data from MDCKII-MDR1 cells indicated that olaparib (1–10 µM) was a substrate for MDR1, with the MDR1 inhibitor ketoconazole reducing the apparent permeability of MDCKII-MDR1 cells to olaparib by 40–62% (Fig. 1A, Supplementary Tables 1 and 2). Whole body autoradiography of male and female rats after single dosing with [^14^C]-olaparib (15 mg/kg, 5 MBq/kg) revealed radioactivity to be undetectable in brain or spinal cord at any timepoint (Fig. 1B, Supplementary Figure 1; Supplementary Table 3). In contrast, PK assessment of olaparib distribution in CD1 nude mice bearing intracranial G7 GBM xenografts revealed tumor penetration at the 2-hour timepoint in all 4 mice, with concentrations varying widely (range, 110–2780 nM; median, 347 nM; Fig. 1C). Olaparib was detected at lower levels in the contralateral (non-tumor bearing) cerebral hemisphere (range, 53–114 nM; median, 84 nM), and plasma levels were approximately 4.5-fold higher than tumor levels (range, 773–2826 nM; median, 1592 nM). Plasma levels fell to approximately 15% by 5 hours (median, 230 nM); olaparib was detectable in one tumor specimen at 5 hours and undetectable in the contralateral hemisphere at 5 and 24 hours.

### Patients

**Fig. 2:**
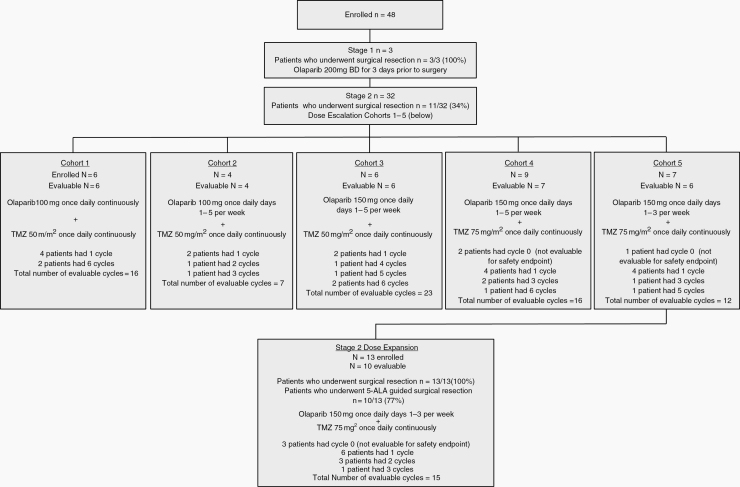
Consolidated Standards of Reporting Trials (CONSORT) diagram of OPARATIC study design and patient disposition.

The OPARATIC study enrolled 48 patients: 3 in stage 1, all of whom underwent surgery; 32 in the dose escalation phase of stage 2, of whom 11 had surgery; and 13 in the dose expansion phase of stage 2, of whom all had surgery. Ten of the 13 dose expansion patients underwent 5-ALA guided resections and tumor margin sampling (Fig. 2). Sixty percent of patients were female and median age was 51 years (range, 18–68). All patients had received radiotherapy as first-line treatment, of whom 47 (98%) had received concurrent chemotherapy.

### Pharmacokinetic Results

**Fig. 3:**
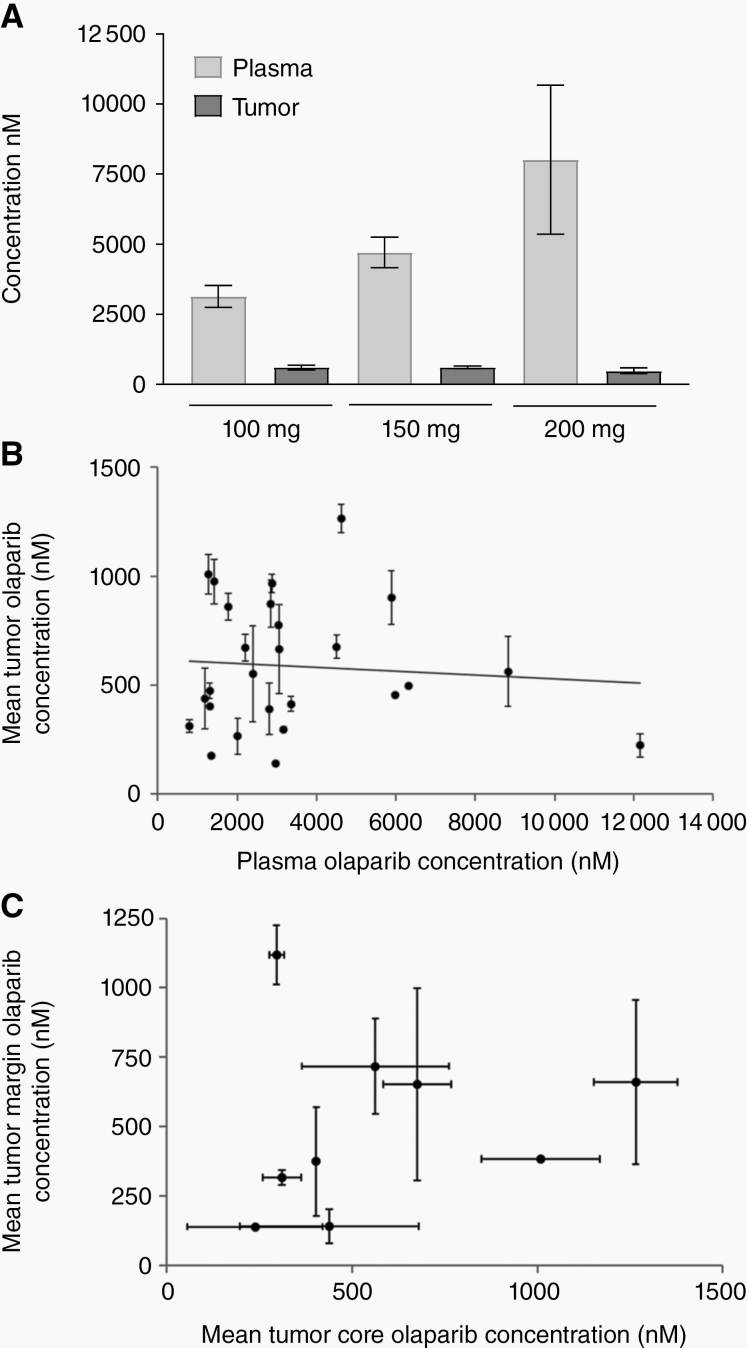
Clinical pharmacokinetic assessment of olaparib. (A) Mean olaparib concentrations in tumor core and plasma samples from patients grouped according to dose of olaparib received in cycle 0 (pre-surgery). 100 mg q.d., *n =* 10; 150 mg q.d., *n =* 35; 200 mg b.i.d., *n =* 3. (B) Mean olaparib concentrations in tumor core specimens plotted against mean plasma olaparib concentrations in 27 patients undergoing surgical resection. (C) Mean olaparib concentrations in tumor margin specimens plotted against mean tumor core concentrations in 9 patients in the dose expansion cohort. All measurements performed by LC-MS.

Olaparib was detected in all 9 samples from the 3 stage 1 patients, triggering progression to stage 2. The mean olaparib concentration in stage 1 samples was 471 nM (range, 164–992 nM, Fig. 3A; 200 mg b.i.d. dose level). From the whole study population, 71 tumor core samples from the 27 surgical patients were of sufficient mass for PK testing. Olaparib was detected in all 71 of these specimens with median concentration 496 nM (range, 97–1374 nM), similar to breast cancer data.^19^ Whereas plasma olaparib levels were broadly dose dependent (Fig. 3A), tumor olaparib concentrations did not correlate with olaparib dose (Fig. 3A) or plasma concentrations (Fig. 3B). Ratios between tumor and plasma olaparib concentrations were highly variable (mean, 0.25; range, 0.01–0.9).

Olaparib was also detected in all 21 tumor margin samples that were of sufficient mass for PK analysis. These samples were obtained from 9 dose expansion patients (Fig. 3C). The median tumor margin olaparib concentration was 512.3 nM (range, 97–1237 nM) and for individual patients the mean ratio between margin and core concentrations was 1.08 (range, 0.32–3.77).

**Fig. 4:**
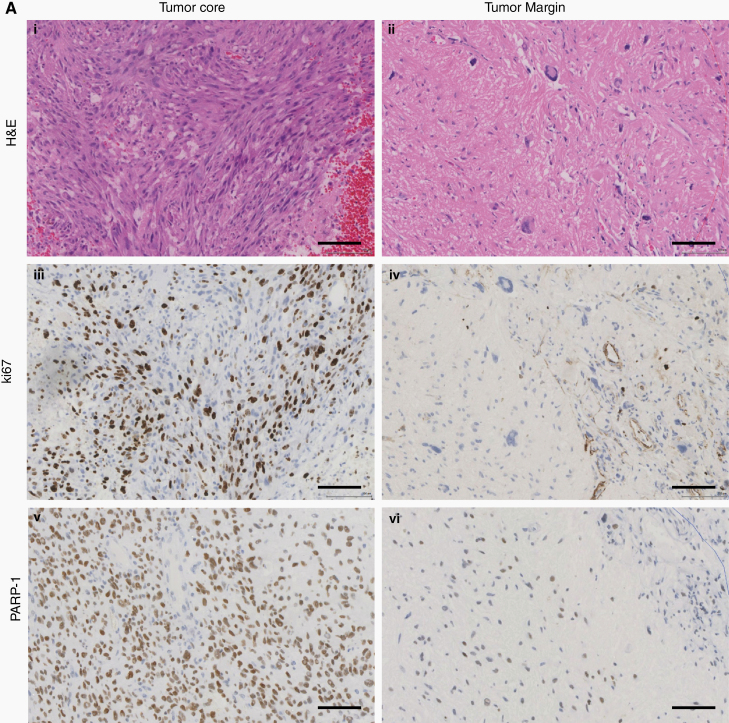

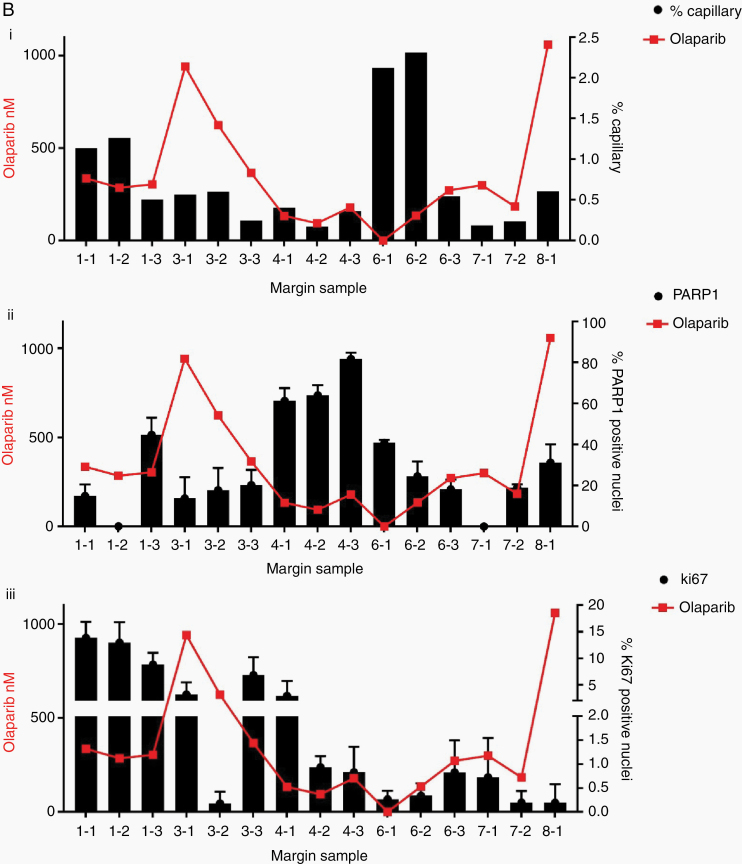
Immunohistochemical analysis of OPARATIC tumor specimens. (A) Representative images of histological sections obtained from a single patient in the dose expansion cohort: H&E (x20) of (i) tumor core and (ii) tumor margin (<3% tumor cell infiltrate); Ki67 immunohistochemistry (x20) of (iii) tumor core and (iv) tumor margin; PARP-1 IHC (x20) of (v) tumor core and (vi) tumor margin showing nuclear immunopositivity. (B) Quantitative analysis of (i) capillary area, detected by CD31 staining; (ii) percentage of PARP-1 positive nuclei; and (iii) percentage of Ki67 positive nuclei in tumor margin specimens from 6 patients in the dose expansion cohort, plotted alongside corresponding olaparib concentrations (red). Stained sections were image captured on Leica Slidepath and image analysis performed using the HALO platform.

Sufficient tumor margin biopsy tissue for IHC analysis was available for 8 patients, of whom 6 had sufficient material for all planned staining protocols. A specialist neuropathologist (K.K.) verified whether H&E stained sections represented genuine tumor margin material and estimated the proportion of tumor cells in each section. IHC staining for 2 putative tumor cell markers PARP-1^20^ and Ki67 was performed where possible, along with CD31 staining to identify blood vessels. Fig. 4A shows representative H&E, Ki67, and PARP-1 staining of tumor core and margin sections from one patient. Supplementary Table 4 shows IHC and olaparib data for each tumor margin specimen. Blood vessels accounted for less than 1% of tumor margin area in each section (mean, 0.67%, range, 0.01–2.30%), and no correlation was observed between olaparib concentrations and blood vessel area (R^2^ = 0.04, *P* = 0.42; Fig. 4Bi), indicating that the olaparib detected in these specimens was not intravascular. Olaparib PK values did not correlate with tumor cell density as measured by 2 surrogate tumor cell markers, PARP-1 and Ki67 (Fig. 4Bii and iii).

### Safety and Tolerability of Concurrent Administration of Olaparib and TMZ

Thirty-nine patients (29 dose escalation, 10 dose expansion) were evaluable for safety. Cohort 1 was expanded to 6 patients because neutropenia (grade 3) and thrombocytopenia (grade 2) necessitated dose reductions in 2 patients. One DLT (grade 3 vomiting) required expansion of cohort 3. One patient in cohort 4 experienced toxic death associated with pancytopenia, septic shock, and renal failure. This schedule was not considered tolerable, because most patients required dose reductions and/or discontinuations. For cohort 5, olaparib was de-escalated to 150 mg once daily (days 1–3 per week) with full dose TMZ (75 mg/m^2^); this was defined as the MTD and confirmed as the RP2D after evaluation in the dose expansion cohort. Of 16 RP2D patients evaluable for toxicity, 7 experienced grade 3–4 hematological toxicities: anemia (3), lymphopenia (7), thrombocytopenia (4), and neutropenia (2). None was complicated by sepsis or bleeding. Additional grade 2 adverse events in this cohort included pruritus (1), nausea (1), and vomiting (1). Adverse events are summarized in Table 1.

### Efficacy

Of 36 patients evaluable for efficacy, 14 (39%; 95% CI: 23.1‒56.5%) remained progression free at 6 months, of whom 9 were in dose escalation cohorts and 5 in the dose expansion cohort. Of 16 evaluable RP2D patients, 10 received 1 cycle, 3 received 2 cycles, 2 received 3 cycles, and 1 received 5 cycles. Since most patients underwent neurosurgery prior to commencing study treatment, measurable disease was uncommon and radiological response was not a study endpoint.

### Imaging Results

Among stage 1 and dose expansion patients, no significant changes in perfusion or permeability parameters were observed between baseline and post-olaparib scans (Supplementary Figure 2). Marked changes were observed in one patient whose histology at resection revealed radiation necrosis rather than recurrent GBM. Average tumor core olaparib concentrations did not correlate with baseline imaging biomarkers, but a significant negative correlation was observed between each patient’s highest olaparib concentration and the K_trans_ value in their baseline scans (R^2^ = 0.39; Supplementary Figure 3A), suggesting that higher olaparib concentrations were associated with low blood flow and/or low endothelial permeability. Maximum olaparib concentration also correlated negatively with fractional plasma volume (R^2^ = 0.28; Supplementary Figure 3B), supporting our assertion that intravascular olaparib was not responsible for tumor drug levels.

### In Vitro Evaluation of Radiosensitizing Efficacy

**Fig. 5:**
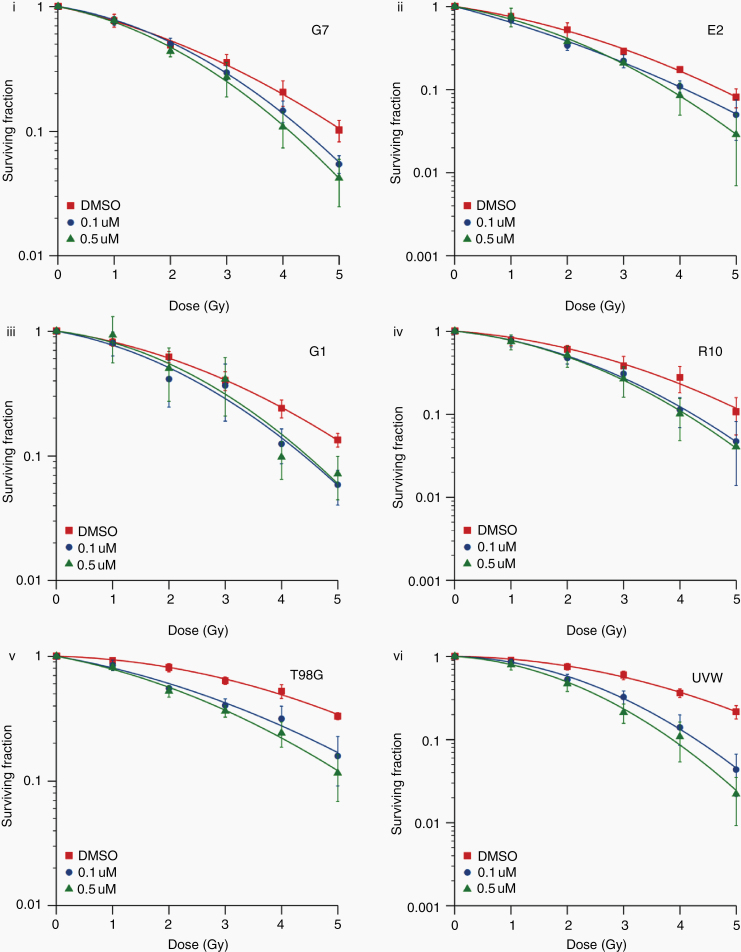
In vitro validation of clinically deliverable olaparib concentrations. The impact of 2 different doses of olaparib on radiosensitivity of 4 primary (i–iv) and 2 established (v–vi) GBM cell lines was measured by clonogenic survival assay. Twenty-four hours after plating, cells were exposed to olaparib or DMSO control then irradiated (1–5 Gy) or sham-irradiated one hour later. Visible colonies containing at least 50 cells were counted 14–21 days after treatment and surviving fraction calculated. The linear quadratic model was fitted using maximum likelihood estimation and integrated to determine the mean inactivation dose (MID) for each experimental condition.

In pilot studies we determined that pharmacodynamic confirmation of PARP inhibition would not be possible in GBM patients because poly(ADP-ribose) (the product of PARP activity) was essentially undetectable in untreated tumor specimens from 5 GBM patients that were obtained from a brain tumor biorepository (Supplementary Figure 4). In the absence of a robust PD biomarker for PARP inhibition, we wanted to assess the likely clinical activity of the olaparib concentrations achieved. The hematological toxicities associated with combined olaparib/TMZ therapy indicate that the most promising role for olaparib in GBM is in combination with radiation therapy. Having observed concentrations above 100 nM in nearly all specimens, and median tumor concentrations of around 500 nM, we evaluated the radiosensitizing effects of 100 and 500 nM olaparib by clonogenic survival assay (Fig. 5). Significant radiosensitization at both concentrations was observed in 6 GBM cell lines, with SER values ranging from 1.1 to 1.7 (Supplementary Table 5) and no dose response apparent within the range tested.

## Discussion

This study demonstrates unequivocally that olaparib penetrates tumor core and tumor margin regions of recurrent GBM, despite preclinical evidence indicating failure to penetrate the intact BBB. The absence of a robust PD biomarker of PARP inhibition together with the infeasibility of obtaining pre/post-olaparib tumor specimens in these patients made it impossible to rigorously assess the clinical activity of the drug concentrations achieved. However, olaparib levels were within the range of 100 to 1000 nM in all specimens, concentrations that achieved significant radiosensitization in all 6 GBM cell lines tested. Combining olaparib with daily low-dose TMZ was safe and reasonably well tolerated, but intermittent olaparib dosing was required to mitigate hematological toxicity. The observed PFS-6 rate of 39% compares favorably with recent clinical trials but was deemed insufficient to support further development of the combination in this population. However, the resoundingly positive PK data have underpinned phase I and II studies of olaparib in combination with radiotherapy ± TMZ in patients with newly diagnosed GBM.^21^ The modest activity of the olaparib-TMZ combination is at least partly attributable to the reduced olaparib dosing that was required to avoid severe hematological toxicity, and in our view should not be taken as evidence of inadequate tumor penetration by olaparib.

This study provides the first direct evidence that a drug showing no penetration of the intact BBB in preclinical models penetrates GBM in patients at clinically meaningful concentrations. Indeed, olaparib penetrated tumor core and margin regions at concentrations similar to those observed in breast cancer specimens.^19,22^ Consistent with studies in other tumor types,^19,22^ no correlation was observed between tumor and plasma concentrations. While variability in the time between olaparib dosing and tumor sampling may have influenced these results, we propose that uptake and retention of olaparib in GBM is determined primarily by tumor vasculature characteristics. This is supported by DCE-MRI data showing an inverse correlation between tumor olaparib concentrations and baseline K_trans_ values. The in vitro radiosensitization data presented here are consistent with an ex vivo study demonstrating >90% inhibition of PARP activity at 50 nM olaparib^23^ and support the concept that meaningful radiosensitization of GBM may be achievable with clinically deliverable olaparib doses. Our study also highlights the need for contemporaneous development of robust PD biomarkers during drug development for GBM and other brain tumors.

To study tumor margin regions, neurosurgeons undertook 5-ALA guided resections to obtain macroscopic clearance of tumor tissue, then sampled adjacent nonfluorescent tissue. These samples were confirmed as tumor margin material by histological analysis: the majority comprised less than 5% tumor cells and only one represented solid tumor. Accurate quantification of tumor cell density was not possible because there are no validated tumor cell markers for IDH wild type GBM, but the surrogate tumor markers PARP-1 and Ki67 supported the histological evaluation. The highly novel finding that tumor margin olaparib concentrations were similar to those in core regions indicates that BBB integrity is compromised even in regions of GBM where tumor cells are sparse and MRI contrast enhancement is not observed. One possible explanation is that co-option of vessels by invading glioma cells enables small numbers of infiltrating tumor cells to disrupt BBB function, as recently demonstrated in orthotopic murine models.^24^

DCE-MRI investigations were performed to assess the impact of olaparib on tumor perfusion and identify candidate imaging biomarkers of olaparib PK. While olaparib did not reproducibly affect DCE-MRI parameters, a negative correlation between maximum tumor olaparib concentration and K_trans_ was observed. We speculate that this reflects increased retention of olaparib within tumors with reduced blood flow and hence reduced drug washout.

Our findings have important implications for preclinical and clinical development of small molecule treatments for GBM and other brain tumors. They illustrate that the preclinical models currently used to measure BBB penetration fail to predict GBM penetration in patients, and motivate us to recommend direct measurement of GBM PK and PD in early phase evaluation of novel agents, in both core and margin regions of these tumors. Finally, our data support the need for more representative preclinical models of the “blood–tumor barrier” to enable rational selection of compounds for development and clinical testing.

## Funding

OPARATIC was sponsored and funded by the Cancer Research UK Centre for Drug Development and supported by the UK National Cancer Research Network. Olaparib was supplied by AstraZeneca.

## Supplementary Material

noaa104_suppl_Supplementary_Material
