# Supplementary material for: Pharmacokinetics, safety, and tolerability of olaparib and temozolomide for recurrent glioblastoma: results of the phase I OPARATIC trial
Source: Neuro Oncol. 2020 Apr 29;22(12):1840–50. doi: 10.1093/neuonc/noaa104 (PMC7746945; doi:10.1093/neuonc/noaa104)
Supplement: noaa104_suppl_Supplementary_Material [file noaa104_suppl_supplementary_material.docx]

**Pharmacokinetics, safety and tolerability of olaparib and temozolomide for recurrent glioblastoma: results of the phase I OPARATIC trial.**

**Supplementary Material**

1. **Supplementary Methods**
2. **Supplementary Tables**

**Supp Table 1** Permeability of control MDCKII and MDR1 transfected MDCKII cells to olaparib.

**Supp Table 2** The MDR1 inhibitor ketoconazole reduces unidirectional transport of olaparib in MDCKII-MDR1 cells.

**Supp Table 3** Concentrations of radioactivity in the tissues of female pigmented rats after a single oral administration of [14C]-olaparib at a nominal dose level of 15 mg/kg body weight.

**Supp Table 4:** Summary of histological, immunohistochemical and pharmacokinetic analyses of tumour margin specimens from patients in the Dose Expansion Cohort.

**Supp Table 5:** Sensitizer enhancement ratio (SER) values for olaparib at 100 and 500 nM in six human GBM cell lines.

1. **Supplementary Figures**

**Supp Figure 1:** Whole body autoradiography of female pigmented rats after treatment with [^14^C]-olaparib.

**Supp Figure 2:** Changes in DCE-MRI parameters pre- and post-olaparib therapy (cycle 0) in 13 individual patients recruited to Stage 1 and the Dose Expansion cohort.

**Supp Figure 3:** Maximum tumour core olaparib concentration correlated inversely with mean fractional plasma volume and median bulk transfer coefficient in eleven patients who underwent two baseline DCE-MRI scans prior to olaparib treatment and subsequent neurosurgical resection.

**Supp Figure 4:** Poly(ADP-ribose) polymer (PAR) immunohistochemistry in U87-MG orthotopic xenograft and patient GBM specimens.

**Supplementary Methods**

**Pre-clinical**

MDCKII cells were obtained from the National Cancer Institute (Amsterdam, The Netherlands).

Monolayers were cultured in Dulbeco’s Modified Eagle Medium (DMEM) supplemented with glucose (4.5 g/L), 10% fetal calf serum, penicillin and streptomycin on semi-permeable filter inserts (Transwell plates 3401, Costar, Cambridge, MA) for three days. Under these conditions MDR1 localises to the apical plasma membrane. [[26](#_ENREF_26)] Monolayer integrity was assessed by transepithelial electrical resistance (TEER) measurement then inserts washed with Hank’s Balanced Salt Solution (HBSS) and transferred to 12-well plates containing HBSS (to measure apical to basal transport, a – b) or [14C]-olaparib diluted in HBSS (dose solution, to measure basal to apical transport, b – a). After addition of dose solution (a – b) or HBSS (b – a) to the apical chamber, samples of apical and basolateral compartments were collected immediately after dosing and after 120 minutes incubation. Three concentrations of 14C-olaparib were tested (0.1, 1 and 10 µM), each in three replicates. Radioactivity was determined by liquid scintillation counting. Inhibition of transport of olaparib or reference substrate vinblastine sulphate was determined in MDCKII-MDR1 cells in the presence of ketoconazole (25 μM). Transport of olaparib or reference substrate cimetidine was determined in MDCKII-BCRP cells. Apparent permeability coefficients (Papp) were calculated:

P_app_ = (dQ/dt)/(A.C0)

in which: dQ/dt = permeability rate (mol/s)

A = surface area filter insert (cm^2^)

C0 = initial concentration (mol/l)

Subsequently parameters of directional transport were calculated:

Efflux ratio = P_app_ b - a /a - b = P_app_ b - a / P_app_ a - b (mean)

MDCKII corrected efflux ratio =

P_app_ b - a /a - b ratio in transfected MDCKII cells

P_app_ b - a /a - b ratio in MDCKII control cells (mean)

For autoradiography studies, animal care and experiments were carried out in accordance with AstraZeneca guidelines. Male and female pigmented rats and female BALB/c nu/nu mice bearing subcutaneous HCT-116 colorectal carcinoma xenografts were treated with 15 mg/kg [^14^C]-olaparib. Animals were culled by asphyxiation, carcasses frozen in hexane and solid carbon dioxide then subjected to whole body autoradiography. Radioactivity concentrations in tissue were quantified from whole-body autoradiograms using a validated image analysis system.

**Clinical trial eligibility criteria**

Eligibility criteria included age ≥18, histological diagnosis of GBM, radiological evidence of recurrence (RANO criteria) after primary treatment with chemoradiotherapy and adjuvant chemotherapy, WHO performance status 0-2 and life expectancy >12 weeks. Laboratory crtieria included hemoglobin ≥10.0 g/dL, neutrophils ≥1.5 x 10^9^/L, platelets ≥100 x 10^9^/L, creatinine clearance ≥50mL/min, bilirubin ≤1.5 upper limit of normal (ULN), alanine aminotransferase (ALT) and aspartate aminotransferase (AST) ≤2.5 ULN. Exclusion criteria included previous chemotherapy for recurrent GBM; radiotherapy, endocrine therapy or immunotherapy within 12 weeks, or chemotherapy or biological therapy within 4 weeks of starting the trial.

**Imaging**

The imaging protocol included four pre-contrast spoiled fast field echo (T1-FFE spoiled gradient echo) sequences with different flip angles (2o, 8o, 15o, 16-20o) for calculation of baseline T1 maps allowing estimation of contrast concentration from observed signal changes and a dynamic, contrast enhanced acquisition series with identical acquisition parameters as the 4th variable flip angle baseline T1 measurement (16-20o), consisting of 100-120 volumes with temporal spacing of 5.1-5.5 seconds (s). Gadolinium-based contrast agent (Gd-DTPA-BMA; Omniscan, GE Healthcare, Oslo, Norway) was injected by pressure injector as a 3 ml bolus at 4 ml/s (dose 0.1 mmol/kg) after acquisition of the seventh image volume, followed by saline flush. Data were analysed using the hybrid model [Li, Jackson Mag Res Med 2003] and in-house software. ROI placement was manual and enhancing pixels were identified through significant change in signal intensity following administration of contrast. In all cases analysis using region of interest metrics was possible with good reproducibility of baseline histograms between baseline scans.

**Imaging biomarkers**

Imaging biomarker calculations used the average of the two baseline measures where applicable.

The following parameters were identified as being of potential significance.

1. Mean and median bulk transfer coefficient ***K*^trans^** (for total enhancing volume, 1/min)
2. Mean and median fractional volume of the extravascular extracellular space ***v*_e_** (for total enhancing volume, unitless)
3. Mean and median contrast agent reflux rate constant ***k*_ep_** (for total enhancing volume, min^-1^)
4. Mean and median **AUCBN(90)** (for total volume, unitless)
5. Mean and median fractional plasma volume ***v*_p_** (for total enhancing volume, mmol/l*s)
6. Total tumour volume and non-perfused volume (mm^3^)
7. Enhancing fraction (unitless)
8. T1 values for enhancing and total tumour volume
9. Mean and median **ADC** (mm^-1)^

Cellular fraction (CF) was added during analysis of the stage 1 data; this was calculated as 1-(v_e_+v_p_) and represents the proportion of each voxel that is not occupied by either blood or extravascular extracellular space (represented by the distribution volume of contrast agent).

**Histopathological methods**

Formalin fixed paraffin embedded (FFPE) tissue from tumor core and margin regions were sectioned (5 μm) and H&E stained with additional immunohistochemical staining for Ki67, PARP-1 (Santa Cruz Sc8007, 1:600) and CD31 (AbCam ab32457, 1:200). Slides underwent antigen retrieval using citrate buffer pH6 (DAKO S2369) and staining was visualised using EnVision HRP (DAKO K4007 & K4011 respectively). Stained sections were image captured on Leica Slidepath and image analysis performed using the HALO platform (Indica labs). PARP-1 is highly expressed in the nuclei of GBM cells and is generally undetectable in normal brain, and the proliferation marker Ki67 discriminates between non-replicating cells of the normal brain and more rapidly proliferating GBM cells. Tumour blood vessels were delineated based on CD31 staining; percentage capillary area was calculated by dividing the capillary area by the total area of the tumor sections studied. Tumor sections from CD-1 nude mice bearing intracranial U87-MG xenografts and GBM patient specimens obtained from Brain Tumour Bank South West (Bristol, UK) were stained for poly(ADP-ribose) (PAR, GeneTex GTX75054, 1:200; antigen retrieval citrate ph9).

| **Olaparib**  **conc**  (µM) | **MDCKII control** | | | **MDCKII-MDR1** | | | **MDCKII**  **Corrected**  **Efflux**  **Ratio**  (SD) |
| --- | --- | --- | --- | --- | --- | --- | --- |
|  | **P_app_**  **a-b**  (SD) | **P_app_**  **b-a**  (SD) | **P_app_**  **b-a/a-b**  (SD) | **P_app_**  **a-b**  (SD) | **P_app_**  **b-a**  (SD) | **P_app_**  **b-a/a-b**  (SD) |  |
| **1** | 1.66  (0.54) | 32.02  (0.85) | 19.33  (0.52) | 0.28  (0.07) | 40.56  (1.74) | 143.97  (6.17) | 7.45  (0.32) |
| **3** | 2.23  (0.41) | 29.67  (1.71) | 13.32  (0.77) | 0.53  (0.07) | 42.70  (2.60) | 80.47  (4.90) | 6.04  (0.37) |
| **10** | 2.46  (0.78) | 28.14  (0.73) | 11.44  (0.29) | 0.58  (0.10) | 39.43  (1.40) | 68.11  (2.42) | 5.95  (0.21) |

**Supp Table 1**

Permeability of control MDCKII and MDR1 transfected MDCKII cells to olaparib. Corrected efflux ratios for 1, 3 and 10 µM olaparib were calculated from mean directional P_app_ values (n=3).

| **Olaparib**  **concentration**  (µM) | **MDCKII-MDR1** | | |
| --- | --- | --- | --- |
|  | **P_app_ b-a**  (SD) | **P_app_ b-a**  **+ ketoconazole**  (SD) | **% of P_app_**  **without ketoconazole**  (SD) |
| **1** | 40.56  (1.74) | 24.12  (1.41) | 59.5  (3.5) |
| **3** | 42.70  (2.60) | 16.34  (1.05) | 38.3  (2.5) |
| **10** | 39.43  (1.40) | 17.06  (0.74) | 43.3  (1.9) |

**Supp Table 2**

The MDR1 inhibitor ketoconazole reduces unidirectional transport of olaparib in MDCKII-MDR1 cells. The effect of 25 µM ketoconazole on permeability of MDCKII-MDR1 cells to 1, 3 and 10 µM olaparib was calculated from mean directional P_app_ values (n=3).

|  |  | nmol equivalents of olaparib per gram of tissue | | | | | |
| --- | --- | --- | --- | --- | --- | --- | --- |
|  |  | 1 hr | 4 hr | 8 hr | 24 hr | 72 hr | 168 hr |
| **Vascular** | Plasma^1^ | 9.69 | 2.11 | 0.180 | 0.046 | 0.009 | ND^2^ |
|  | Blood | 2.44 | 0.729 | 0.171 | BLQ^3^ | BLQ | BLQ |
| **Metabolic/**  **excretory** | Kidney cortex (outer) | 9.52 | 3.16 | 1.49 | 0.248 | 0.202 | BLQ |
|  | Liver | 112 | 49.3 | 14.7 | 2.23 | 0.604 | 0.164 |
|  | Small intestine mucosa | 60.5 | 19.1 | 7.93 | 1.19 | BLQ | BLQ |
|  | Large intestine mucosa | 12.1 | 11.1 | 39.8 | 2.10 | BLQ | BLQ |
| **CNS** | Choroid plexus | 0.618 | 0.228 | BLQ | BLQ | BLQ | BLQ |
|  | Brain | BLQ | BLQ | BLQ | BLQ | BLQ | BLQ |
|  | Meninges | 0.847 | 0.500 | BLQ | BLQ | BLQ | BLQ |
|  | Pineal body | 2.13 | 0.318 | 0.248 | BLQ | BLQ | BLQ |
|  | Spinal cord | BLQ | BLQ | BLQ | BLQ | BLQ | BLQ |

**Supp Table 3**

Concentrations of radioactivity in the tissues of male pigmented rats after a single oral administration of [^14^C]-olaparib at a nominal dose level of 15 mg/kg body weight.

**Supp Table 4**

Summary of histological, immunohistochemical and pharmacokinetic analyses of tumour margin specimens from patients in the Dose Expansion Cohort.

|  | **SER**  (95% CI)  **100nM** | **P-value** | **SER**  (95% CI)  **500nM** | **P-value** |
| --- | --- | --- | --- | --- |
| **G7**  MGMT methylated | **1.105**  (1.018 - 1.200) | 0.013* | **1.188**  (1.029 - 1.390) | 0.012* |
| **E2**  MGMT methylated | **1.237**  (1.065 - 1.441) | 0.008* | **1.221**  (1.076 - 1.378) | 0.009* |
| **G1**  MGMT methylated | **1.225**  (1.01 - 1.513) | 0.019* | **1.119**  (0.828 - 1.654) | 0.19 |
| **T98G**  MGMT unmethylated | **1.42**  (1.128 - 1.883) | 0.006* | **1.584**  (1.464 - 1.71) | 0.0005* |
| **UVW**  MGMT methylated | **1.478**  (1.28 - 1.726) | 0.0001* | **1.695**  (1.407 - 2.098) | 0.0001* |
| **R10**  MGMT methylated | **1.266**  (0.991 - 1.565) | 0.0307* | **1.281**  (0.966 - 1.721) | 0.037* |

**Supp Table 5**

Sensitizer enhancement ratio (SER) values for olaparib at 100 and 500 nM in six human GBM cell lines. SER values and 95% confidence intervals calculated from mean inactivation dose (MID) values derived from clonogenic survival curves shown in Figure 5.


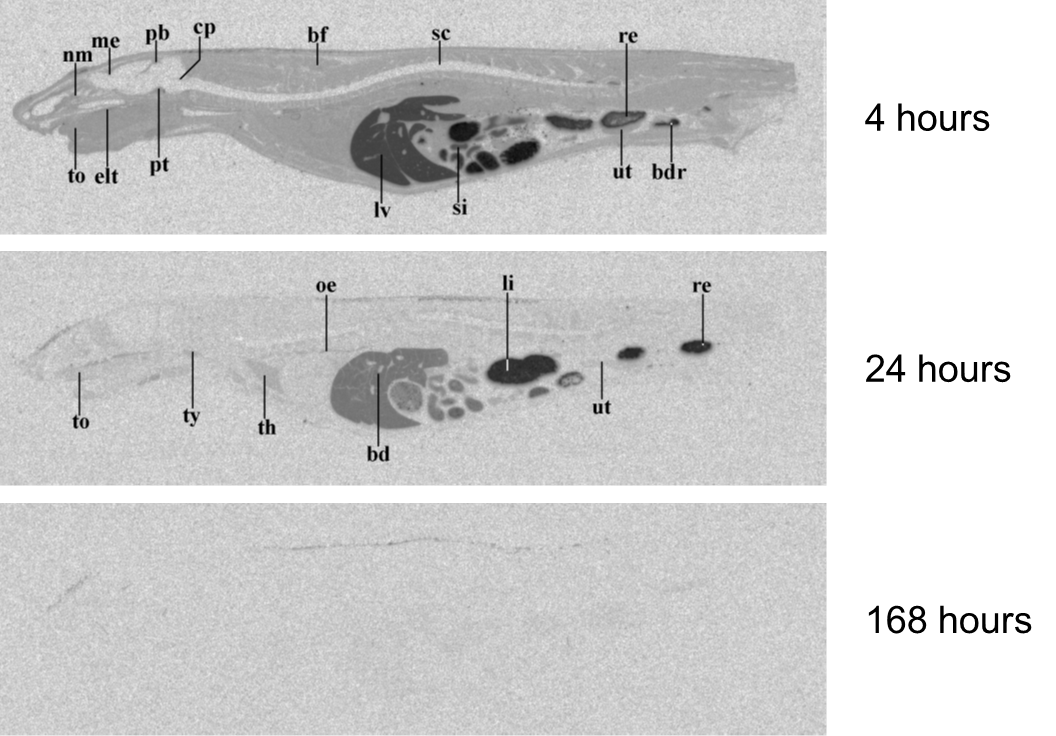


**Supp Figure 1**

Single oral doses of 15 mg/kg [^14^C]-olaparib were administered to female pigmented rats which were subsequently culled, sectioned and subjected to whole body autoradiography at the timepoints shown. Radioactivity was excluded from the central nervous system in all animals at all timepoints.


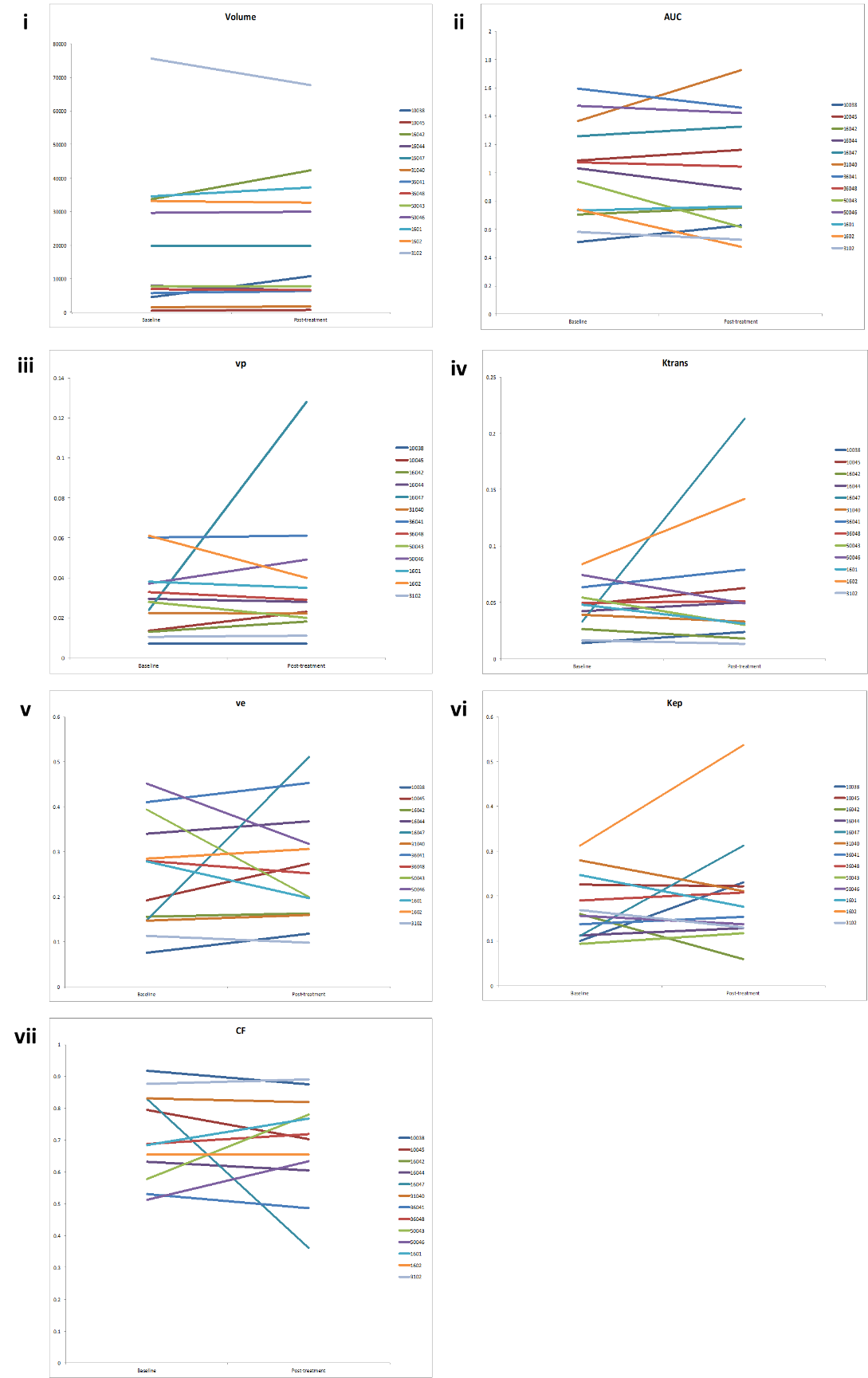


**Supp Figure 2**

Changes in DCE-MRI parameters pre- and post-olaparib therapy (cycle 0) in 13 Stage 1 and Dose Expansion patients. Baseline measurements are the average of two baseline scans (one patient had a single baseline scan). (i) Total tumour volume, (ii) area under the gadolinium concentration curve normalised with plasma input function (AUCBN(90)), (iii) mean fractional plasma volume (vp), (iv) median bulk transfer coefficient (Ktrans), (v) median fractional volume of the extravascular extracellular space (ve), (vi) contrast agent reflux rate constant (Kep), (vii) cellular fraction (CF). Patient 16-047 had radiation necrosis rather than recurrent GBM. No effects of olaparib on DCE-MRI parameters were observed in patients with confirmed recurrent GBM.


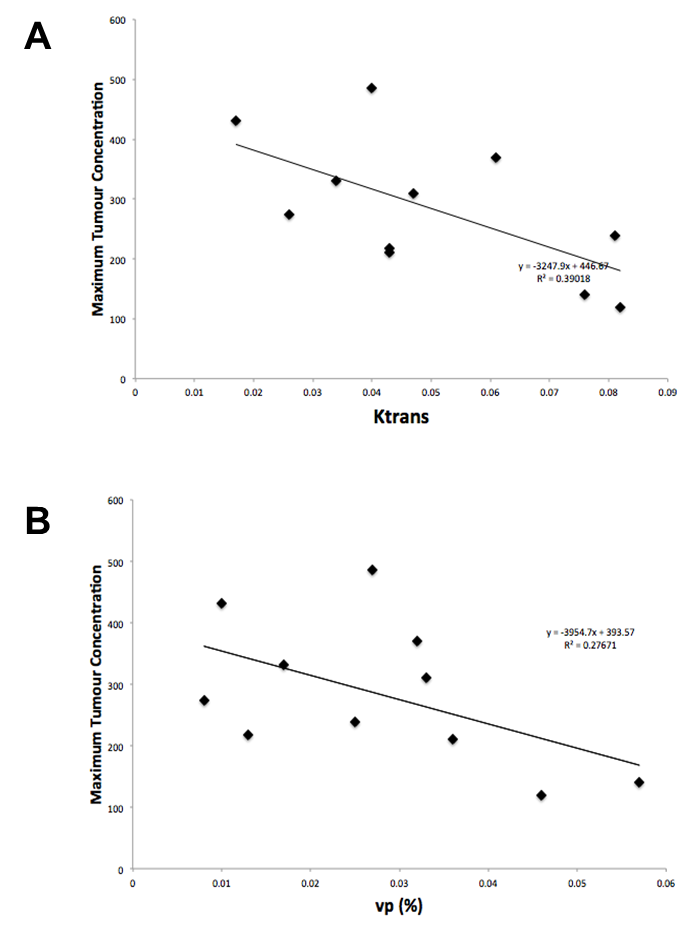


**Supp Figure 3**

Maximum tumour core olaparib concentration correlated inversely with mean fractional plasma volume (v_p_, R^2^=0.39), and median bulk transfer coefficient (K_trans_, R^2^=0.28) in eleven patients who underwent two baseline DCE-MRI scans prior to cycle 0 olaparib treatment and subsequent neurosurgical resection. Patients received different olaparib doses depending on cohort.


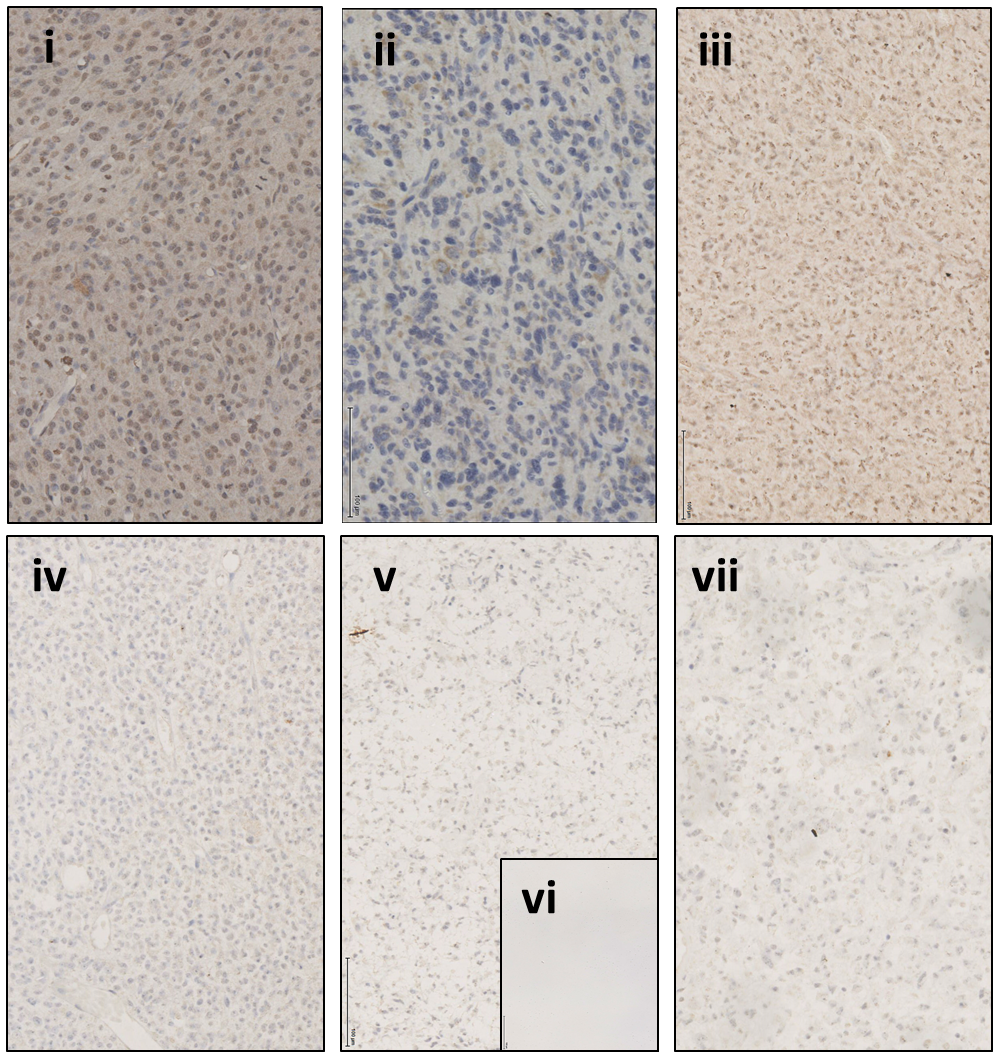


**Supp Figure 4**

Representative images of histological sections displaying immunohistochemistry for poly(ADP-ribose) (PAR)**. (i)** U87MG mouse xenograft (perfuse-fixed, positive control). **(ii-vii)** Representative PAR staining of five different human GBM samples. **(vi)** Human GBM sample with no primary antibody (negative control).
